# Supplementary material for: The structure of the endogenous ESX-3 secretion system
Source: eLife. 2019 Dec 30;8:e52983. doi: 10.7554/eLife.52983 (PMC6986878; doi:10.7554/eLife.52983)
Supplement: Supplementary file 1. [file elife-52983-supp1.docx]

Supplementary file 1. Model Refinement Statistics.

Final refinement validation for all amino acids which were modeled de novo.

| Model Refinement | |
| --- | --- |
| Total amino acids | 3,156 |
| All-atom clashscore | 13.49 |
| Ramachandran outliers | 0.00% |
| Ramachandran allowed | 5.39% |
| Ramachandran favored | 94.61% |
| Rotamer Outliers | 0.00% |
| C β Deviations | 0% |
| Cis-proline | 0% |
| Cis-general | 0.00% |
| Twisted proline | 0% |
| Twisted general | 0.00% |
