# Supplementary material for: The structure of the endogenous ESX-3 secretion system
Source: eLife. 2019 Dec 30;8:e52983. doi: 10.7554/eLife.52983 (PMC6986878; doi:10.7554/eLife.52983)
Supplement: Supplementary file 3. [file elife-52983-supp3.docx]

**Supplementary file 3.** Buried surface area.

Buried surface area between subunits as measured by PISA.

| Interaction interfaces | | |
| --- | --- | --- |
| Monomer 1 | Monomer 2 | Buried surface area (Å2) |
| EccDbent - protomer i | EccDextended - protomer i | 3154 |
| EccDbent - protomer ii | EccDextended - protomer ii | 3116.3 |
| EccDextended - protomer i | EccB - protomer i | 1370.2 |
| EccDextended - protomer ii | EccB - protomer ii | 1295.6 |
| EccE - protomer i | EccDbent - protomer i | 1157.2 |
| EccE - protomer ii | EccDbent - protomer ii | 1122.4 |
| EccDextended - protomer i | EccC - protomer i | 881.9 |
| EccDextended - protomer ii | EccC - protomer ii | 825.1 |
| EccDbent - protomer i | EccC - protomer i | 779.9 |
| EccDbent - protomer ii | EccC - protomer ii | 746.6 |
| EccB - protomer i | EccC - protomer i | 635.7 |
| EccE - protomer i | EccDextended - protomer i | 608.5 |
| EccE - protomer ii | EccDextended - protomer ii | 600.1 |
| EccB - protomer i | EccC - protomer ii | 576.1 |
| EccB - protomer ii | EccC - protomer ii | 502.3 |
| EccB - protomer i | EccDextended - protomer ii | 482.1 |
| EccB - protomer i | EccDbent - protomer i | 297.2 |
| EccB - protomer ii | EccDbent - protomer ii | 288.6 |
| EccB - protomer i | EccB - protomer ii | 255.6 |
| EccC - protomer i | EccC - protomer ii | 213.6 |
| EccB - protomer i | EccDbent - protomer ii | 168.9 |
| EccDextended - protomer i | EccB - protomer ii | 56.6 |
| EccDextended - protomer i | EccDextended - protomer ii | 49.9 |
| EccDextended - protomer i | EccC - protomer ii | 40.1 |
| Cross-protomer interaction interfaces | | |
| EccB - protomer i | EccB, EccC, EccD - protomer ii | 1482.7 |
| EccC - protomer i | EccC - protomer ii | 213.6 |
| EccDextended - protomer i | EccB, EccC, EccDextended - protomer ii | 146.6 |
